# Assessing Perinatal Psychiatric Morbidity: Implications for Maternal Mental Health Care in Italy

**DOI:** 10.32872/cpe.15117

**Published:** 2025-08-29

**Authors:** Giulia Ciuffo, Marta Landoni, Chiara Ionio

**Affiliations:** 1 CRIdee, Trauma Research Unit, Department of Psychology, Faculty of Psychology, Catholic University of the Sacred Heart, Milan, Italy; 2 CRIdee, Department of Psychology, Faculty of Psychology, Catholic University of the Sacred Heart, Milan, Italy; Friedrich-Alexander-Universität Erlangen-Nürnberg, Erlangen, Germany

**Keywords:** post-traumatic stress disorder, birth, recommendations, policy, perinatal, screening

## Abstract

**Background:**

Traumatic births impact women’s long-term health, family dynamics, and healthcare systems, underscoring the need for prevention and effective interventions. Despite Italy's universal healthcare, perinatal mental health services and guidelines, especially for childbirth-related PTSD (CB-PTSD), remain underdeveloped. This study aims to investigate the prevalence of CB-PTSD, postpartum depression (PPD), and anxiety in Italian women 6-12 weeks postpartum, and assess the impact of comorbidities on mother-child bonding.

**Method:**

The study was part of a broader longitudinal research that involved 175 Italian mothers 6-12 weeks postpartum, recruited from birthing centers. Participants completed measures for childbirth-related PTSD (City BiTS-IT), depression (EPDS), anxiety (PSAS-IT), and mother-child bonding (PBQ).

**Results:**

Prevalence rates were 1.1% for CB-PTSD, 18.6% for depression, and 30.2% for anxiety. Depression was significantly associated with anxiety (χ^2^(1, *N* = 159) = 9.131, *p* = .003) and CB-PTSD (χ^2^(1, *N* = 171) = 11.689, *p* < .001). Hierarchical regression showed that depression and general PTSD symptoms significantly impaired mother-child bonding, explaining 36.3% of the variance *(R*^2^ = 0.363).

**Conclusion:**

The findings highlight the prevalence and complexity of perinatal psychiatric morbidity, emphasizing the critical need for comprehensive assessment tools tailored to the Italian context. These results contribute to a deeper understanding of maternal mental health challenges during the perinatal period.

## Highlights

- 1.1% met full diagnostic criteria for childbirth-related PTSD, 68.4% had one or more symptoms.
- CB-PTSD was associated with depression and comorbidity impaired mother-infant bonding.
- Screening for CB-PTSD is often neglected. This paper offers a validated screening tool.

Pregnancy and childbirth bring significant physical, psychological, and social changes. The perinatal period, which extends from the beginning of pregnancy to one year postpartum, is a complex and vulnerable time that presents various challenges for both women and men in the transition to parenthood (Parfitt & Ayers, 2014).

Research suggests that approximately 1 in 3 births are perceived as psychologically traumatic (Alcorn et al., 2010) and meta-analyses show that 4% of women who give birth later develop childbirth-related PTSD (CB-PTSD), while clinically significant CB-PTSD symptoms are observed in 17% of women (Heyne et al., 2022).

The mother-child bond, the emotional and cognitive connection between a mother and her child, is of central importance for the child's development and the mother's well-being. Previous research has shown that poor parent-child attachment has been associated with impaired emotional, behavioural and cognitive development in the child, as well as affective disorders in adulthood (Dekel et al., 2020). Although previous studies have shown how postpartum depression and anxiety can affect mother-infant bonding (Dekel et al., 2020), less is known about the potential impact of birth-related PTSD.

Traumatic birth experiences and the resulting CB-PTSD symptoms can cause considerable distress and have a significant long-term impact on the health of women, their babies and their families. It is now well established that maternal CB-PTSD can affect both maternal and spousal relationships (Garthus-Niegel et al., 2018; Hairston et al., 2018) and can also lead to long-term negative effects on child behaviour and development (e.g. Cook et al., 2018).

Traumatic births can also affect medical staff (Uddin et al., 2022), resulting in significant costs to healthcare systems and potential economic consequences for society (Bauer et al., 2014). Therefore, the prevention of traumatic births and CB-PTSD is a global priority. This mandate is in line with the United Nations Millennium and Sustainable Development Goals for better health for women, mothers and children (United Nations DESA, 2022), the World Health Organisation’s call for dignified and respectful maternity care for every woman (WHO, 2015) and the Council of Europe and European Parliament’s resolution on the importance of women’s sexual and reproductive rights (European Parliament, 2022). Although a recent systematic review of the cost-effectiveness of interventions for perinatal disorders, including CB-PTSD, concluded that screening, psychological or social support and specialised treatment programs are all effective and cost-effective interventions to address these issues (Verbeke et al., 2022), screening for CB-PTSD is often neglected among perinatal mental disorders (PMDs). Currently, only a handful of countries have developed strategies to prevent traumatic births and treat CB-PTSD (Thomson et al., 2021). Even in the World Health Organisation’s recommendations on maternal and newborn care for a positive postnatal experience (WHO, 2022), only depression and anxiety are mentioned in the few lines dedicated to mental health. The lack of clear policies or guidelines means that many people have limited access to the services they need (Sperlich et al., 2017).

Since 1978, the Italian National Health Service (Servizio Sanitario Nazionale, SSN) has guaranteed universal access to healthcare. The central government determines the national core benefits package and provides funding for the regional healthcare systems. The 19 Italian regions and two autonomous provinces are responsible for funding, planning and providing services at the local level, supported by a network of around 100 local health authorities (Signorelli et al., 2020). Significant differences exist in the provision of healthcare services across the country (Cicchetti & Gasbarrini, 2016). In Italy, there is no specialised service for perinatal mental health, so mental health care for women of childbearing age is provided by mental health departments (MHDs; Grussu et al., 2020). Family Care Centres (FCCs), which are integrated into SSN community services, provide free support to women during pregnancy and in the postnatal period and focus on the early identification of perinatal mental health problems (Grussu et al., 2020). A national guideline for perinatal mental health care is currently not available (Lega et al., 2024). In addition, healthcare providers often have limited training in selecting the most appropriate screening tool and determining the appropriate cut-off point for specific time periods (Cena et al., 2020). Despite these findings, recent research suggests that the prevalence of perinatal mental disorders (PMDs) in Italy is similar to other European countries (e.g. Camoni et al., 2023; Ciuffo et al., in preparation), so there is currently a lack of knowledge about how the national mental health service supports women during the perinatal period. The first study providing insights into the availability of evidence-based best practices for perinatal mental health (PMH) within the Italian mental health service dates back to 2024 (Lega et al., 2024). However, recommendations on prevention and screening for CB-PTSD are still lacking.

The predominant perinatal mental disorders that occur in women during pregnancy and postpartum are depressive syndromes and anxiety syndromes (Cena et al., 2020). Comorbidity of these disorders is common, reaching 40% in some studies (i.e., Misri & Swift, 2015). Research indicates that CB-PTSD is often comorbid with depression and anxiety disorders (i.e., Horesh et al., 2017), which increases the likelihood of postpartum psychiatric problems in women with CB-PTSD. Of women diagnosed with PTSD, 65% also exhibit symptoms of postpartum depression (PPD), while 22% of women with PPD also exhibit symptoms of PTSD (Söderquist et al., 2006). Due to the frequent overlap of these syndromes, it is important to consider how these factors interact with maternal mental health (Grisbrook & Letourneau, 2021). The failure to include recommendations for CB-PTSD screening in existing guidelines may be due to a failure to recognise the prevalence and impact of CB-PTSD on maternal quality of life and family functioning, and the need for validated screening tools (Grisbrook & Letourneau, 2021).

Against this background, the present study aims to investigate the prevalence of CB-PTSD, PPD, and anxiety, along with their comorbidity, in a sample of Italian women 6–12 weeks postpartum, as well as to assess the impact of potential comorbidities on mother-child bonding.

## Materials and Method

### Ethics

Ethical approval was granted by the ethics committees of the Catholic University of the Sacred Heart (reference: 05-22) and the S. Giuseppe Hospital in Milan (reference: 550/2022). Before starting the survey, participants had to read an information sheet and electronically sign a consent form with Qualtrics. Data collection was conducted according to the principles of the Declaration of Helsinki and in accordance with the ethical guidelines of the IRB.

### Procedure and Participants

The City BiTS-IT was included in a wider international project, the International Survey of Childbirth-related Trauma (INTERSECT), which has been registered at: https://www.researchregistry.com/browse-the-registry#home/registrationdetails/5ffc7453702012001b80a58c/

In Italy, the INTERSECT project was conducted by the Trauma Psychology Research Unit of the Catholic University of the Sacred Heart in Milan. The Italian component followed a longitudinal design with three waves of data collection. In the initial phase (T1), participants were recruited in birth centers, hospitals or clinics and completed the questionnaire via Qualtrics. They were then contacted 6-12 weeks after the birth (T2) and again 6 months after the birth (T3) for a follow-up survey. No social media or other online methods were used during recruitment to avoid self-selection bias. The inclusion criteria were: (i) participants in the third trimester of pregnancy, (ii) aged 18 years or older, (iii) with informed consent and (iv) proficient in Italian.

### Measures

Alongside socio-demographic and obstetrical information, participants completed the following measures.

#### The City Birth Trauma Scale (City BiTS)

CB-PTSD was assessed using the validated Italian version of the City Birth Trauma Scale (BiTS; Ayers et al., 2018; Ciuffo et al., in preparation). The BiTS assesses PTSD symptoms based on DSM-5 criteria, including exposure to traumatic stressors, intrusive symptoms, avoidance, negative cognitions and mood, hyperarousal, and dissociative symptoms. The scale also assesses symptom occurrence, duration, distress, impact on daily life and possible physical causes. The total score resulting from criteria B-E ranges from 0 to 60. You can find more detailed information about the BiTS in the Supplementary Materials (Ciuffo et al., 2025S).

#### The Edinburgh Postnatal Depression Scale (EPDS)

Maternal depression was assessed using the Italian version of the Edinburgh Postnatal Depression Scale (EPDS; Cox et al., 1987; Benvenuti et al., 1999). This self-report questionnaire assesses the severity of maternal depression during the postpartum period. Scores range from 0 to 30, with a score between 9 and 10 indicating clinically significant depression. The internal reliability was ω = 0.84. Detailed information on the evaluation can be found in the Supplementary Materials (Ciuffo et al., 2025S).

#### The Postpartum Specific Anxiety Scale (PSAS)

The mothers' anxiety was assessed using the Italian version of the Postpartum Specific Anxiety Scale (PSAS; Fallon et al., 2016; Ionio et al., 2023). This questionnaire comprises 51 items that were developed to assess anxiety symptoms in the postpartum phase. The scale measures four areas of anxiety. Scores range from 51 to 204, with a threshold score of 112 indicating clinically significant anxiety. Further details about the PSAS can be found in the Supplementary Materials (Ciuffo et al., 2025S).

#### The Postpartum Bonding Questionnaire (PBQ)

Maternal and paternal attachment was assessed using the Italian adaptation of the Post-Birth Attachment Questionnaire (PBQ; Brockington et al., 2001; Busonera et al., 2017). The questionnaire comprises four subscales and uses a six-point Likert scale to assess attachment difficulties. Further information about the PBQ can be found in the Supplementary Materials (Ciuffo et al., 2025S).

### Statistical Analyses

The analyses were carried out with IBM SPSS Statistics Version 29.0. Descriptive statistics (means, standard deviations, frequencies) were calculated to summaries the characteristics of the sample and the prevalence rates of psychiatric disorders. Recommended cut-offs were used to analyse the rates of depression and anxiety. Diagnostic criteria were used to calculate the prevalence of PTSD. Participants were categorised based on their scores. Comorbidity of CB-PTSD, PPD and anxiety was analysed using cross-tabulations and chi-square tests to determine the proportion of women suffering from multiple disorders simultaneously. Hierarchical regression analyses were performed to examine the influence of CB-PTSD, PPD and anxiety (independent variables) on mother-infant attachment (dependent variable). Previous mental disorders, complications for the mother or child during labour and mode of delivery were included as covariates in the model.

## Results

The sample consisted of 175 Italian women with a mean age of 32.75 years (*SD* = 4.68). The majority were either married or cohabiting (93.7%), and 60.6% had attained higher education. In terms of employment, most participants were engaged in professional, office, or technical occupations, while a small percentage were unemployed or homemakers. All participants were primiparous, and the majority (74.0%) had a vaginal birth, with 34.7% experiencing minor maternal complications during delivery. Infant complications were reported in 11.1% of cases. Additionally, 28.2% had experienced a previous pregnancy loss, and 12.8% reported uncertainty regarding a past diagnosis of mental illness. Table S1 (Supplementary Materials – see Ciuffo et al., 2025S) summaries the main demographic characteristics and obstetric information of the mothers.

Table 1 shows the prevalence rates of psychiatric disorders and the distribution of specific diagnostic criteria to provide a comprehensive understanding of symptom patterns and diagnostic thresholds.

**Table 1:** Distribution of Psychiatric Disorder Diagnostic Criteria and Prevalence Rates

Characteristic	Percentage
CB-PTSD Criterion A: exposure to traumatic stressor	14.3
CB-PTSD Criterion B: Re-experiencing	58.6
CB-PTSD Criterion C: Avoidance symptoms	12.6
CB-PTSD Criterion D: Negative cognitions and mood	35.6
CB-PTSD Criterion E: Hyperarousal	61.1
CB-PTSD Criterion F: Duration	53.1
CB-PTSD Criterion G: Distress or impairment	57.5
Having one or more symptoms of trauma	68.4
Full criteria for CB-PTSD met	1.1
Depression above the cutoff	18.6
Anxiety above the cutoff	30.2

*Note.* The total sample for this table included 175 participants.

The cross-tabulation analysis between postnatal depression and anxiety showed a significant correlation. Of the 175 participants, 159 gave valid responses (90.9%), while 16 were missing (9.1%). Of the women with valid data, 84 met the criteria for either depression or anxiety: 46 women met the criteria for anxiety but not for depression, 27 women met the criteria for depression but not for anxiety, and two women met the requirements for both. The remaining nine cases were women who did not fulfil the criteria for either disorder. The chi-square test for independence showed a statistically significant relationship between depression and anxiety, χ^2^(1, *N* = 84) = 9.131, *p* = .003. This was further confirmed by the likelihood ratio, χ^2^(1, *N* = 159) = 11.258, *p* < .001, and Fisher’s exact test, *p* (bilateral) = .002. These results suggest that the presence of depression is significantly associated with an increased likelihood of anxiety in postpartum women.

The association between CB-PTSS and anxiety was examined in 158 valid cases (90.3%) and 17 missing cases (9.7%). The distribution was as follows: 33 women met criteria for neither CB-PTSS nor anxiety, 19 women met criteria for anxiety but did not have CB-PTSS, 78 women met criteria for CB-PTSS but not anxiety, and 28 women met criteria for both. The chi-square test showed no statistically significant association between PTSS and anxiety, χ^2^(1, *N* = 158) = 1.711, *p* = .191. The likelihood ratio, χ^2^(1, *N* = 158) = 1.680, *p* = .195, and Fisher’s exact test, *p* (bilateral) = .200, also showed no significant association. This suggests that the presence of PTSD symptoms related to childbirth was not significantly correlated with anxiety in this sample.

For the relationship between CB-PTSS and postnatal depression, there were 171 valid cases (97.7%) and 4 missing cases (2.3%). The results of the contingency table were as follows: 52 women did not meet criteria for either CB-PTSS or depression, two women met criteria for depression but not CB-PTSS, 87 women met criteria for CB-PTSS but not depression, and 30 women met criteria for both. The chi-square test showed a statistically significant association between CB-PTSS and depression, χ^2^(1, *N* = 171) = 11.689, *p* < .001. The likelihood ratio, χ^2^(1, *N* = 171) = 14.541, *p* < .001, and Fisher’s exact test, *p* (bilateral) < .001, confirmed this significant association. These results suggest that women with postnatal depression are more likely to experience PTSD symptoms related to childbirth.

Finally, a hierarchical regression model was developed to examine the effects of individual and comorbid disorders on mother-infant attachment 6-12 weeks postpartum. In Step 1, depression was used as a predictor of attachment, in Step 2 anxiety was integrated, in Step 3 the CB-PTSS (birth-related symptoms and general symptoms) and in Step 4 the covariates were added. Depression was found to be a significant predictor of disturbed mother-infant attachment and explained 28% of the variance. This suggests that higher levels of postpartum depression are associated with poorer attachment. The addition of anxiety did not significantly improve the model, suggesting that anxiety is not a strong predictor of mother-infant attachment in this sample. When birth-related and general PTSD symptoms were added, the model improved significantly, with general PTSD symptoms being a particularly strong predictor. This model explained 34.8% of the variance, suggesting that trauma-related symptoms play a significant role in mother-infant attachment. The inclusion of additional covariates (such as type of birth and pre-existing mental health diagnoses) did not further improve the model, suggesting that these variables do not have a significant impact on mother-infant attachment in this context. The following table (Table 2) summaries these results.

**Table 2:** Hierarchical Regression Predicting Mother-Child Bonding

Predictor	*B*	*SE B*	β	*t*	*p*
Step 1 (*R*^2^ = 0.280)					
Depression	1.073	0.137	.529	7.836	< .001
Step 2 (*R*^2^ = 0.280)					
Depression	1.072	0.138	.529	7.741	< .001
Anxiety	-0.002	0.032	-.004	-0.055	.957
Step 3 (*R*^2^ = 0.348)					
Depression	0.740	0.167	.365	4.435	< .001
Anxiety	0.015	0.031	.031	0.465	.643
Birth-related PTSD symptoms	-0.257	0.187	-.100	-1.374	.171
General PTSD symptoms	0.592	0.148	.337	4.002	<.001
Step 4 (*R*^2^ = 0.352)					
Depression	0.753	0.171	.371	4.412	< .001
Anxiety	0.015	0.032	.031	0.461	.645
Birth-related PTSD symptoms	-0.255	0.193	-.099	-1.323	.188
General PTSD symptoms	0.565	0.152	.322	3.708	< .001
Vaginal delivery	1.350	2.466	.062	0.547	.585
Assisted vaginal delivery	-0.257	3.517	-.006	-0.073	.942
Emergency CS	2.276	2.999	.079	0.759	.449
Pre-existing Diagnosis	-0.623	1.943	-.022	-0.321	.749

*Note.* Mode of delivery includes four categories: vaginal delivery, assisted vaginal delivery, emergency CS, and planned CS. Dummy coding was applied, with planned CS as the reference category. Pre-existing mental health diagnosis is a binary variable (yes/no).

## Discussion

Perinatal mental disorders are widespread and represent a major health problem. Awareness, prevalence rates and treatment of these mental disorders vary greatly from country to country (i.e., Dikmen-Yildiz et al., 2017). The present study aimed to investigate the prevalence and comorbidity of CB-PTSD, PPD and anxiety in a sample of Italian women 6-12 weeks postpartum. In addition, the effects of these potential comorbidities on mother-infant bonding were investigated.

In terms of prevalence rates, depression and anxiety were similar to previous literature (i.e., Hahn-Holbrook et al., 2018), while CB-PTSD was slightly lower than the observed global pooled prevalence (Heyne et al., 2022), albeit similar to previous studies (i.e., Dikmen-Yildiz et al., 2017). Although only 1.1% of women met the full diagnostic criteria for CB-PTSD, the vast majority of mothers (68.4%) had one or more CB-PTSS, demonstrating how prevalent this pathology is in this population. In addition, the results confirmed the co-occurrence of childbirth-related PTSD symptoms and depression in 17.54% of the sample, similar to previous research in this field (Dekel et al., 2020), suggesting that women with depression are more likely to have CB-PTSS and vice versa. In our sample, we found no significant association between CB-PTSS and anxiety. However, previous studies (Dikmen-Yildiz et al., 2017) have found increased comorbidity between these disorders 6 months postpartum. This suggests that the co-occurrence of these disorders becomes more evident later in the postpartum period as conditions become more structured. Longitudinal studies are needed to investigate these associations throughout the postpartum period. The presence of comorbidities indicates that if depression or PTSD is suspected, it is important to look at other symptoms for treatment purposes. It may also be beneficial to consider these disorders as part of a continuum of stress and to use the term ‘postnatal mood disorders’ proposed by Matthey et al. (2003), which includes birth-related PTSD. There is extensive evidence that early assessment of PPD can predict maternal attachment difficulties up to one year postpartum (e.g. Kasamatsu et al., 2020), but there is considerably less research examining the relationship between attachment and other psychopathologies such as CB-PTSD. Women with comorbid mental disorders are more likely to have impaired functioning and experience higher levels of stress than women with only one disorder (e.g. Horesh et al., 2017). Although PPD is widely recognised as a significant risk factor for poor attachment, the relationship between CB-PTSD and attachment is less well understood. Some studies have found a clear association between CB-PTSD and impaired attachment (Parfitt et al., 2014), while others have not observed such an association (Nakić Radoš et al., 2020). This inconsistency may be due to differences in the definition of CB-PTSD, the methods used to measure it, and the consideration of other factors, such as comorbid depression (Davies et al., 2008). Our findings suggest that the effects of CB-PTSD on attachment may vary from person to person. In particular, symptoms of CB-PTSD, especially when combined with depression, appear to disrupt emotional bonding between mothers and their infants. In contrast, birth-related PTSD symptoms alone appear to have less of an impact on attachment. This is consistent with recent research that distinguishes between birth-related and more general PTSD symptoms (e.g. Nakić Radoš et al., 2020), with general PTSD symptoms showing a stronger association with impaired attachment.

As mentioned above, the lack of recommendations for CB-PTSD screening in existing guidelines may be due to a lack of awareness of the prevalence and impact of CB-PTSD on maternal quality of life and family functioning, as well as the need for validated screening tools (Grisbrook & Letourneau, 2021). Indeed, a recent systematic review (Ciuffo et al., 2025) highlighted that a challenge in screening for CB-PTSD is the limited availability of validated questionnaires specifically designed to assess this disorder.

The lack of customised instruments also makes it difficult to determine the true prevalence of the disorder, which remains poorly researched and overlooked in Italian maternity facilities (Ciuffo et al., in press). Screening needs to take into account organisational factors that affect implementation as well as the availability of valid measurement tools to accurately identify the disorder. To date, the City Birth Trauma Scale (City BiTS; Ayers et al., 2018) is the only self-report specifically designed to measure CB-PTSD according to DSM-5 diagnostic criteria and validated for the Italian population (Ciuffo et al., in press). The City BiTS-IT has good reliability and strong psychometric properties. It is a quick and straightforward instrument, making it highly recommended for the early detection of childbirth-related PTSD (Ciuffo et al., in press).

In 2019, an international consortium of researchers and clinicians specialising in traumatic birth and CB-PTSD was established to advance knowledge and practise in this area (European Commission Cooperation in Science and Technology (COST Action grant CA18211). This group developed recommendations for practise, research and policy (Ayers et al., 2024). In terms of research, the use of “CB-PTSD validated, diagnosis-based instruments is required. To determine diagnoses and prevalence, cut-off values need to be established and adapted to different cultural settings”. Substantial evidence of the prevalence and consequences of CB-PTSD will facilitate the assessment of the economic burden associated with this condition, thus providing a sound rationale and financial motivation for prioritising prevention and intervention efforts in areas that are currently under-supported in health systems worldwide (Ayers et al., 2024).

### Strengths and Limitations of the Study

This study has several limitations. First, it relies on self-reported measures, which may be subject to response biases such as social desirability or memory distortions, which could affect the accuracy of reported symptoms and experiences. On the other hand, the questionnaires are easy to administer and score, making them ideal for early assessment and prevention of PMDs. Second, the cross-sectional design limits the possibility of establishing causal links between psychiatric disorders and disrupted mother-child attachment. While our results suggest significant associations, the direction of these associations remains unclear. For example, it is unclear whether psychiatric symptoms directly affect attachment or whether attachment difficulties exacerbate maternal psychological distress. Longitudinal data would provide a more comprehensive understanding of the trajectory of maternal mental health and attachment over time. Future research should use longitudinal studies to track the development of symptoms, identify potential mediators or moderators in this relationship, and assess the long-term impact on maternal and infant well-being. Second, the cross-sectional design may limit the ability to draw causal conclusions. Future research should consider longitudinal studies to better understand possible pathways of comorbidity of these disorders as well as their long-term effects on family functioning. Finally, study participants were recruited in birth centers, hospitals and clinics without using social media or other online methods. While this approach helps to reduce self-selection bias, it may also exclude women who do not seek inpatient care or are less familiar with these healthcare facilities. This study uses validated measures, ensuring a reliable and standardised assessment. Furthermore, to the best of the authors’ knowledge, this is the first study to investigate the prevalence and impact of CB-PTSD and its comorbidities in the Italian context, providing new insights into perinatal mental health in Italy.

### Conclusions

The high rates of mental disorders after birth emphasise several important implications for policy and clinical practice. Effective perinatal screening is crucial for identifying women with mental health problems. Early detection and intervention can reduce the likelihood of postnatal mental disorders. Timely treatment of women affected by these disorders is important to ensure their mental well-being and that of their families and to prevent these disorders from becoming chronic. National and international guidelines on maternal mental health are needed to raise awareness of perinatal mental health problems, including CB-PTSD, and to present evidence-based, practical strategies for detection, prevention and treatment. Future research and policy statements should also include men and/or other birth partners.

This review adds to the literature highlighting the great need to prioritise women’s mental health in both research and clinical practice, and reflects calls for greater attention to this area within the psychological community (e.g. Ayers et al., 2024). Our study, which focuses on the prevalence of perinatal psychiatric disorders and their impact on mother-infant attachment, provides a foundation for future research and policy decisions aimed at improving women's mental health. This could be a first step in addressing a historically overlooked aspect of public health and ensuring that women receive the support they need during this critical stage of life.

Overall, this research has shown that a significant number of women in Italy suffer from significant depression, anxiety, CB-PTSD or a combination of these conditions during the perinatal period. Healthcare providers and policy makers should recognise the significant psychological distress experienced by Italian women during the perinatal period and the potential long-term impact on women and their families.

## Supplementary Materials

Supplementary File 1 – Table 1 and Measures (see Ciuffo et al., 2025S): This Supplementary Material includes a table summarizing the demographic characteristics and obstetric information of the 175 Italian mothers who participated in the study. It provides details such as maternal age, region of residence, marital status, occupation, educational attainment, and various obstetric variables, including mode of delivery and maternal/infant complications during birth.

Additionally, the Supplementary Material offers detailed information on the self-report questionnaires used in the study: the City Birth Trauma Scale (City BiTS), the Edinburgh Postnatal Depression Scale (EPDS), the Postpartum Specific Anxiety Scale (PSAS), and the Postpartum Bonding Questionnaire (PBQ). Each measure is described in terms of its structure, scoring, and internal reliability.



CiuffoG.
LandoniM.
IonioC.
 (2025S). Supplementary materials to "Assessing perinatal psychiatric morbidity: Implications for maternal mental health care in Italy"
[Additional information]. PsychOpen. 10.23668/psycharchives.21076
PMC1291847541727815

## Data Availability

The raw data supporting the conclusions of this article, as well as the code used for data analysis and the materials employed during the study are available from the authors upon reasonable request. These resources will be provided directly to researchers who contact the corresponding author, without undue reservation.

## Related Versions

This research was conducted as part of the International Survey of Childbirth-Related Trauma (INTERSECT, www.intersectstudy.org).

## Reporting Guidelines

This paper was written following STROBE reporting guidelines (Strengthening the Reporting of Observational Studies in Epidemiology).

## Social Media Accounts

*Chiara Ionio:* LinkedIn

## Preregistration

This study is part of the broader international project *International Survey of Childbirth-related Trauma (INTERSECT)*, which was preregistered on the Research Registry. The preregistration includes the general study aims and methodological framework of the international survey and is publicly accessible at: https://www.researchregistry.com/browse-the-registry#home/registrationdetails/5ffc7453702012001b80a58c. However, the analysis of the Italian subsample and the specific research questions addressed in the present article were not preregistered.

## Appendix: List of Abbreviations

CB-PTSD – Childbirth-related Posttraumatic Stress Disorder
CB-PTSS – Childbirth-related Posttraumatic Stress Symptoms
PPD – Postpartum Depression
PMDs – Perinatal Mental Disorders
PMH – Perinatal Mental Health
MHDs – Mental Health Departments

## References

[sp1_r1] Ciuffo G. Landoni M. Ionio C. (2025 S). Supplementary materials to "Assessing perinatal psychiatric morbidity: Implications for maternal mental health care in Italy" [Additional information]. PsychOpen. 10.23668/psycharchives.21076 PMC12918475 41727815

